# Determinants of access to general practice in a shared care model for people living with HIV: a qualitive study of patients’ perspectives in an Australian rural community

**DOI:** 10.1186/s12875-023-02142-1

**Published:** 2023-09-06

**Authors:** Juliet Cunningham, Jodie Bailie, Sherridan Warner, Ashleigh Condon, Daniel Cheung, Ariane Minc, Simone Herbert, Natalie Edmiston

**Affiliations:** 1 Faculty of Medicine and Health, The University of Sydney, Sydney, Australia; 2 University Centre for Rural Health, The University of Sydney, Lismore, Australia; 3 School of Public Health, The University of Sydney, Sydney, Australia; 4 Northern New South Wales Sexual Health Service, North Coast Public Health, Mid North Coast Local Health District, Lismore, Australia; 5 School of Medicine, Western Sydney University, Campbelltown, Australia

**Keywords:** Access, Shared care, Rural health, General practice, Primary care, HIV

## Abstract

**Background:**

Improved management of human immunodeficiency virus (HIV) has resulted in improved life expectancy for people living with HIV and an ageing population with a significant comorbidity burden. Shared care models, involving the co-ordinated liaison between general practitioners and specialist physicians, have been advocated for in Australia to provide comprehensive care. People living with HIV in rural areas have reduced access to general practice and therefore shared care. This study explores the perspectives of people living with HIV on the barriers and enablers to accessing shared care in an Australian rural setting.

**Methods:**

In this qualitative study, semi-structured interviews were conducted with adults living with HIV who either resided in or accessed care in a rural area of Australia. Interviews were conducted via video conferencing, phone or face-to-face. Transcripts were imported into NVivo, coded and analysed in alignment with a conceptual framework of healthcare access defined by Levesque and colleagues.

**Results:**

Thirteen interviews were conducted in total. Participants’ narratives demonstrated the substantial influence of accessibility to general practice on their ability to engage in effective shared care. Challenges included the perception that general practitioners would not provide additive value to participants’ care, which restricted the ability to both seek and engage in the shared care model. Healthcare beliefs, expectations and experiences with stigma led participants to prioritise the perceived interpersonal qualities of specialist care above a shared care system. Access to shared care was facilitated by continuity of care in general practice but logistical factors such as affordability, transport and availability impacted the ability to access regular high-quality healthcare.

**Conclusions:**

Navigating patient priorities and anticipated stigma in general practice within the resource limitations of rural healthcare were barriers to effective shared care. General practitioners’ ability to build rapport and long-term relationships with participants was instrumental in the perception of valuable care. Strategies are required to secure continuity of care with interpersonally skilled general practitioners to ensure provision of quality primary care for people living with HIV, which can be supported by specialist physicians in a shared care model.

## Background

Pharmacological advancements in treating human immunodeficiency virus (HIV) have significantly reduced mortality rates in people living with HIV (PLWH) [1]. The current population of PLWH is larger and older than in the past [2] with a disproportionately high comorbidity burden compared to age-matched peers [3]. As approaches to HIV management have evolved to become increasingly long term, a greater role for primary care is emerging in PLWH to include screening, prevention, and management of non-HIV-related and HIV-related comorbidities. Unfortunately, PLWH experience more barriers to accessing primary care resulting in lower rates of cancer screening and increased hospital admissions when not engaged with regular primary care [4, 5]. Improving access to primary care for PLWH may improve health outcomes and quality of life [6–8].

The evolving roles and responsibilities of the healthcare system in supporting long-term care of PLWH have been outlined in a consensus statement released by Lazarus et al*.* [9]. Addressing barriers of access to care was identified as a key concern in future healthcare planning, including the improved implementation of models of care that connect PLWH with primary care [9]. In accordance with this recommendation, the New South Wales (NSW) Health HIV strategy 2021–2025 proposed a state-wide shared care model to optimise the link between community and specialist healthcare [10]. Shared care is a team-based model of care involving a coordinated and communicative arrangement between General Practitioners (GPs), specialists and other members of one’s healthcare team [10] which has been widely propounded internationally [5, 9, 11–13].

In rural Australia, HIV care is predominantly accessed through publicly funded multi-disciplinary sexual health clinics [14], similar to speciality-based care models conducted internationally [5, 15]. These clinics are separate from GPs who are the predominant providers of primary care in Australia. GP consultations are either fully funded (bulk billed) or partially subsidised by the government at the discretion of the GP and the practice. PLWH in rural settings have varied engagement with GPs and have historically accessed general medical care at sexual health clinics more than those in inner urban areas [14]. Multiple barriers to GP and general healthcare engagement have been identified for PLWH, including anticipated stigma [12, 16, 17], confidentiality risks [5, 12, 18], appointment unavailability [13, 18], cost [16, 17, 19] and lack of HIV-knowledge based care [5, 12, 14, 16]. Financial stressors, transport challenges [2] and heightened confidentiality concerns [16] are particular challenges to rural Australian populations. As interventions are more successful when barriers and enablers to their utilisation are identified and addressed [20], analysis of the barriers to shared care in a previously specialist-led, rural context is necessary for effective healthcare provision.

**Fig. 1:**
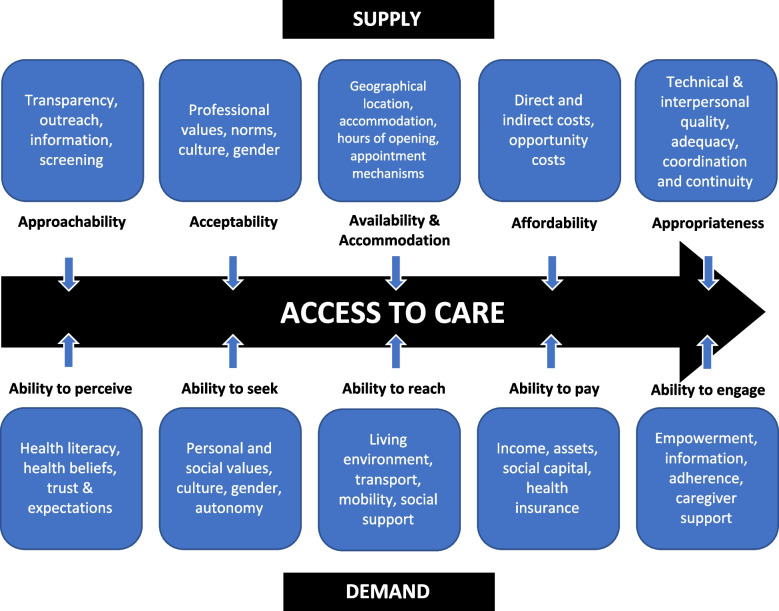
Adapted framework of healthcare access conceptualised by Levesque et al*.* [21]

The access framework conceptualised by Levesque et al*.* [21] describes five determinants of access related to both the service seeker and service provider (Fig. 1). The use of this framework has been substantiated as a tool to comprehensively analyse influencers to accessing healthcare [22]. Numerous studies have used this framework in the context of primary health care access specifically [23], particularly for vulnerable populations [24–26]. The framework organises the stages of access from perceiving a service to consumer engagement and represents the role of both the individual and population, as well as the providers, services, and health systems, in one’s ability to access care. To date, a holistic, qualitative analysis of the factors influencing access to a shared care model of healthcare has not been conducted in Australia or internationally for PLWH. By utilising the Levesque framework, this study aims to elucidate the key barriers and enablers to accessing shared care identified by PLWH in a rural setting.

## Methods

### Setting

This study was conducted in 2022 in Northern NSW, a region of rural Australia. In 2021, 532 residents in this health district were prescribed antiretroviral therapy for HIV [10]. This area contains the Northern NSW sexual health clinics, which provide patients with HIV specialist, nursing, and social work services free of charge and are accessible via self or GP referral. In Northern NSW, general non-HIV related healthcare may be accessed at local GPs including “s100 GPs” who are specially accredited in prescribing antiretroviral therapy. At the time of this study there were few s100 GPs in Northern NSW according to a registry [27], which is typical for rural areas. A study of 329 PLWH attending the Northern NSW sexual health clinics found that slightly more than half had a GP involved in their care [28] despite multimorbidity being common in the clinic attendees, with 25% reporting 3 or more additional chronic health conditions [29]. Support services for PLWH in Northern NSW include community based organisations providing a range of social and practical support services for PLWH [30, 31].

### Study design

This was a qualitative study involving semi structured interviews and deductive thematic analysis using a framework approach [32]. An a priori decision was made to utilise each of the Levesque [21] determinants as the framework for the analysis. This study was completed as a subproject within a larger qualitative study investigating patient-perspectives on HIV shared care in Northern NSW. Design and reporting of the study were guided by the Consolidated criteria for Reporting Qualitative research guidelines [33].

### Participants

PLWH were recruited through a combination of convenience and purposeful sampling. Initial invitation was via waiting room posters and social media and open to any interested PLWH. Direct verbal invitation was made by a social worker, author AM, to additional PLWH who were less engaged with these aforementioned sites and less likely to see the study invitations, to include a variety of perspectives. Snowballing was also utilised. Participant criteria included being over 18 years old, able to speak and understand English, ability to consent, and receiving HIV care or residing in Northern NSW. All participants were provided a $30 gift card as remuneration.

### Data collection

The semi-structured interview covered a range of topics, including factors influencing current healthcare engagement with a focus on GPs, understanding of the shared care model, perceptions of interprofessional communication in shared care, and perceived value of care (Table 1). The interview guide was developed by NE, an HIV specialist, in consultation with AM and SH, a social worker and doctor in the field respectively, and reviewed by a colleague who was a PLWH.

**Table 1:** Semi structured interview guide

Would you like to tell me a little about how long you have been living with HIV and where you have been living during this time?How long have you been living in Northern NSW?Besides HIV, what other health conditions are you dealing with? *Probes – heart disease, mental health, liver disease, memory problems?*What different health care providers do you currently see? *Probes – HIV/SH doctor, HIV/SH counsellor, nurse, GP, other counsellor, physiotherapist, chiropractor, dentist?*How would you rate your current health?* Probes – Very poor, poor, fair, good, very good*What other supports do you have to help you live with HIV? *Probes – Bobby Goldsmith Foundation, ACON*
Section 1 What has been your experience of accessing GPs? *Probes – regular GP vs non regular, number of different GPs* If yes, what do you like about having GP care? What problems do you experience in using a GP? *Probes – benefits, transport, time, cost, information/communication* If no, would you like to have a GP? What would be good things about having a GP? What has prevented you from using a GP? – *Probes – perceiving need, suitability of GPs, transport, time, cost, information/communication*
Section 2 Have you heard the term shared care? What does it mean to you? *Provided definition – By shared care we mean that health practitioners are working together to provide your health care in a co-ordinated manner, usually involving a GP, a specialist, and other providers* Do you have shared care for your HIV? If yes, can you describe what that looks like for you? In the last few years, Northern NSW HIV services have moved to encourage shared care where possible. How has this affected/impacted you? To what degree do you feel like you have a choice in how you receive care? What do you think sexual health services can do to improve shared care with GP in the future?
Section 3 What health care do each of the different health practitioners provide for you? How do you decide what health care issues each practitioners helps you with? How do the various health care providers communicate with each other? Do you have concerns about the communication between health care providers? *Probes—too much communication, not enough, consent for sharing of information?* What is your role in your care? If you see a number of health services, who helps co-ordinate or navigate that process?
Section 4 What are the most valuable aspects of the care that you receive from your care providers? Are there aspects of care that you receive, or have received, that you think is of little or low value? *Probes – clinical visits, blood tests* If yes, why do you think low value care is happening? Does having more than one person involved in your care contribute to receiving low value care? How does your care contribute to your wellbeing?

Interviews were conducted one-on-one by either DC, AC, JC, or SW (one male, three female), who were medical students at the time of the study conducting a university research project. Interviewers received training on qualitative methods, interviewing skills, HIV healthcare in NSW and destigmatising care prior to commencement. The interviewers had an interest in sexual health and the training processes allowed for reflection on personal circumstances and beliefs of the interviewers and their interaction with the study. Interviewers had not met the participants prior and were not engaged in the participants’ healthcare team.

Interviews were performed either face-to-face at two Northern NSW sexual health clinics, via video conferencing (PEXIP software) or over the phone, without the use of field notes. Interviewers critically reflected on their own assumptions to promote a heightened ability to listen to the participants’ stories as openly as possible. Interviews averaged 52 min (range 26–88 min), were audio-recorded and transcribed verbatim by the interviewers. Participants were given the option to review their transcript prior to analysis but not repeat the interview. A priori thematic saturation was achieved following the first ten interviews, indicated by all determinants obtaining instances of data [34].

### Data analysis

The lead author (JC) read the interview transcripts numerous times, making reflective notes throughout. JC and JB developed codes a priori to guide the analysis based on the access determinants espoused by Levesque et al*.* [21] and as described in Fig. 1. Initially, one author (JC) engaged in a deductive process in which broad codes were applied to the data after each interview. These codes included each of the supply and demand determinants of the access framework (Fig. 1). Following completion of the interviews, the data were revisited, and the coding was checked by JC and NE.

Interviewers met regularly from the initial interviews to identify and discuss data and the direction of subsequent interviews, with analysis commencing during the interview stage. The data within the codes finalised by JC were then further reviewed with NE and preliminary themes identified based on the access determinants. This was conducted by either combining codes to better represent the predominant factors influencing each access determinant or ascribing findings to their most significant determinant when relevant to more than one. These decisions were made in consultation with authors NE, SW, AC and DC.

Early findings were presented to healthcare professionals working in Northern NSW sexual health clinics and themes were discussed and refined. Minor adjustments were required to achieve good concordance in the categorisation, analysis, and interpretation of the data. Finally, all authors checked if the findings were consistent with their perceptions and understanding based on their experience as interviewers for this study and as health professionals. The robustness of the findings was enhanced by multiple review cycles, the application of constant comparison techniques [35] (whereby each interpretation and finding was compared with existing findings as they emerged from the data analysis process) and multiple discussions between the interviewers and NE. Participants were not consulted regarding the findings of the study. The qualitative data management program, NVivo was used for coding, searching, and organising transcript data [36].

## Results

Thirteen interviews were completed with most participants male and over 50 years old (Table 2). No participants identified as Aboriginal or Torres Strait Islander. Most participants had long term HIV diagnoses and had been residing in Northern NSW for over 10 years. All participants had at least one comorbidity with the majority experiencing between 1 and 3 comorbid conditions. No participants requested amendments to their transcripts.

**Table 2:** Interview respondent characteristics

Respondent characteristics	Interview respondents
*Gender*	
Male	11
Female	1
Other	1
*Age bracket (years)*	
50–59	6
60–69	7
*Length of time living in Northern NSW (years)*	
< 2	1
2–10	2
> 10	10
*Length of time living with HIV (years)*	
< 2	0
2–10	1
> 10	12
*Number of comorbidities*	
0	0
≤ 3	8
> 3	5
*Subjective assessment of health*	
Poor	4
Fair	2
Good	7

Findings are presented in accordance with the dimensions of access proposed by Levesque et al. [21]. Illustrative quotes are provided in Table 3.

**Table 3:** Dimensions of access and subthemes with illustrative quotes

Dimensions of access [21]	Subtheme	Illustrative quotes
‘Approachability’ and ‘ability to perceive’	Health beliefs and expectations	*It’s not ‘cause I hate doctors, it’s just, with my friends dying back in the day with AZT, I became very distrusting of the whole system. And that’s where people are today, distrust in the system. (Interviewee #3)*
		*The specialist… he’s good on the infectious diseases side of things, but anything else… he’s said to me, “I wouldn’t have a clue, I’d probably kill ya. But I can do… this is my specialty”. Where your GP is an allrounder. (Interviewee #7)*
‘Acceptability’ and ‘ability to seek’	Anticipated stigma affecting health-seeking behaviours	*I’ve had hideous things said to me about, from doctors… and I never went back, you know?… [and I approach healthcare now] kind of apprehensive. (Interviewee #6)*
		*I never disclosed my status to anyone, any doctor or dentist or whatever in Sydney… [and when I moved to] Northern Rivers, I found a doctor … he was, to my surprise, quite friendly and we go through the whole health history, and he definitely showed me that the mentality here… he made me feel good! So, I disclosed to him my status and he was quite good… I’m quite happy that I chose him as my GP. (Interviewee #2)*
‘Availability and accommodation’ and ‘ability to reach’	Distance to appointments	*The thing about the Northern Rivers, all the transport’s been a thing for me. I’ve got a car now… but I think the Northern Rivers is a sort of satellite, you know, so I guess travelling was one thing to get to them. My previous GP was in (locality) and uh, I was living out there for a little while but once I returned to my house here, it just made it harder to get to… so yeah, it’s mainly been about location, I think is the one [barrier]. (Interviewee #5)*
	Appointment availability	*I guess finding a GP was challenging because it was, you know, a lot of closed books and practices… so that was challenging… (Interviewee #10)*
‘Affordability’ and ‘ability to pay’	Affordable, quality care	*I have to go to the local bulk billing place and the doctors change there so often that um, you now, you get used to one doctor who knows your history or is a good doctor and then you may have a not-so-great doctor who, who knows your story and then they might have underlying prejudices towards you… (Interviewee #3)*
		*I really appreciate, you know, the thoroughness, because as a human, we’re really complicated and health is a complicated issue that needs to be addressed with professionalism and thoroughness and I get that… that is like, hugely valuable to me. (Interviewee #8)*
‘Appropriateness’ and’ability to engage’	Technical competency	*I don’t know if this is a thing with GPs or what, but it seems that the specialists inform GPs, but the GPs don’t inform the specialists… that’s what I feel like I’m doing… I think it should come from the doctor… I don’t see why a GP caring for me should not be involved in shared care with my HIV doctor, I think that’s a bit strange. (Interviewee #4)*
	Interpersonal quality	*I love my GP, like love my GP… [I have been seeing her] going on 2 years?… I like her personability and personality. I like the fact that I’m, cause medicine is something that I, I have some medical training, so I’m, you know, always researching around things, so I like the fact that she’s open to discussion around things… I feel like it’s a dialogue, it’s not like “I’m your doctor, here’s your prescription, go away”. (Interviewee #5)*
	Health efficacy	*When I first started seeing [HIV specialist], he had a concept which I still hold true and dear to my heart, which is that I am an integral part of my healthcare team, whereas other people view their healthcare as oh, their healthcare professionals do that. I have to be an integral part of the team to affect the best outcome… I’m not just an unwitting participant… I can’t be passive; I have to be absolutely proactive for my own outcome. (Interviewee #5)*

### ‘Approachability’ and ‘ability to perceive’

#### Health beliefs and expectations

Many participants held the belief that GPs did not provide additive value to their care and perceived them as having a subsidiary role to HIV specialists. One participant expressed viewing their GP as only a necessary step toward accessing their HIV specialist referral. Another participant commented that they required continuous prompting by their specialist before they engaged with a GP. One participant described preferring a specialist-only model despite “knowing” a GP was beneficial. While many participants valued physical wellbeing as a healthcare outcome, there was significant silence regarding the positive health outcomes of including a GPs’ medical expertise in care. Only one participant explicitly acknowledged the broader scope of expertise provided by GPs and how that could positively impact their care.

Participants’ health beliefs and expectations also influenced their ability to perceive a need for shared care. Many valued their HIV specialist highly, but some demonstrated scepticism in other doctors including GPs, using derogatory terms such as “quacks”, or describing a general dislike for GPs. Others recounted previous negative experiences with early HIV care regimes and the resultant distrust for the healthcare system. Amotivation also led some to resist shared care, displaying signs of chronic illness burnout exhibited by expressions of disinterest in their health, difficulty planning healthcare decisions, or wanting to self-discontinue medical treatment. A significant proportion also believed a regular GP was not needed due to their current health stability. This was contrasted by a few participants who highly valued the role of GPs in their wellbeing. Some attributed this to a sense of security gained by having a family GP as a child.

### ‘Acceptability’ and ‘ability to seek’

#### Anticipated stigma affecting health-seeking behaviours

Past stigmatised experiences in healthcare were a significant barrier to currently accessing care for many participants. Some participants described negative feelings following healthcare interactions, mainly with GPs, that led to them feeling vulnerable or apprehensive when subsequently seeking care. Some participants cited anticipated stigma as the reason for resisting GP care, describing fears of confidentiality breaches, judgement, or reluctance to disclose their HIV status. Early rapport building was suggested to facilitate access in those anticipating stigma. One participant described a friendly, inquisitive initial GP consultation leading them to disclose their HIV status despite never previously feeling comfortable to do so with a healthcare provider. Another participant described having better clinical experiences with doctors they could relate to due to less fear of stigma. A participant also suggested they would be more encouraged to use a GP if they either advertised an interest in HIV care or if HIV specialists recommended them specifically as being anecdotally low risk for stigma.

### ‘Availability and accommodation’ and ‘ability to reach’

#### Distance to appointments

Most participants with cars or centralised care around the regional hubs found transportation a nonissue. Inability to travel to appointments was one participant’s sole barrier to accessing healthcare and another stated that distances to GPs sometimes prevented them from attending appointments at all. Constant relocation of GPs and some participants between local towns was cited as a barrier to access and meant either travelling large distances to maintain their GP relationship or sacrificing their continuity of care. Distance was also the reason that one participant was more likely to attend the hospital’s emergency department than a GP for a recurrent health condition. A few participants found that telehealth encouraged shared care access by reducing travel, but one participant added the caveat that it was only successful when prior rapport had been established.

#### Appointment availability

Many participants highly valued appointment availability and found it significantly lacking with local GPs. Some participants described inability to book new GP appointments or obtain continuous appointments at a practice leading to increased travel and cost related to care. Lack of availability also increased some patients’ health anxieties and anticipated stigma as they repeatedly required new initial GP appointments without having prior rapport, making them less likely to engage. One patient noted lack of availability as the primary negative distinction between their care rurally and in urban locations.

### ‘Affordability’ and ‘ability to pay’

#### Affordable, quality care

Due to financial circumstance, some participants sacrificed quality care for affordability. A low density of bulk billing GPs was identified as a barrier by multiple participants leading some to either avoid GPs or preference HIV specialist care due to financial concerns. Other participants noted that the bulk billing practices available were more rushed with inflexible appointment lengths, had greater staff turnover and placed less value on rapport, increasing their discomfort and perceived risk of stigma.

### ‘Appropriateness’ and ‘ability to engage’

#### Technical competency

Participants considered shared care appropriate when they perceived technical competency in their GP. Many participants valued doctors’ technical expertise in their care and were satisfied with the HIV-related knowledge-based care available at GPs but often found consultations rushed and lacking thoroughness. This was viewed as a dismissal of participants’ complex health needs and as a result, some described feeling less confidence and security in their care. When time was taken to complete thorough consultations with any healthcare professional, participants viewed this as higher quality care and a facilitator of trust and likelihood of returning.

Participants identified effective interdisciplinary communication as important for competent shared care. When participants perceived good communication between practitioners, they felt consultations were more effective, more confidence in their overall care or experienced less unnecessary testing. One participant believed communication was unidirectional from the HIV specialist to the GP, and not reciprocated. Another described the GP as responsible for coordinating communication with the specialist, but the latter generally “picking up the slack”. One participant, who resided in a town on a state border, identified a unique barrier to those accessing healthcare in two states, which have distinct state health departments. They highlighted the lack of infrastructure for efficient communication between doctors in the separate healthcare systems as a barrier to shared care.

#### Interpersonal quality

Establishment of doctor-patient trust and rapport was integral to disclosure and continuity of care. Evident across all interviews was the importance of personability and rapport-building in clinicians, and personalised interactions for the participants’ needs, anxieties, or interests. In some cases, participants described their doctor as an important social and emotional support in their life. This was almost exclusively in reference to HIV specialists, but when present in GP care, it was a strong indicator for encouraging continuity. GP care was also facilitated by a holistic approach to the participant’s health, friendly initial consultations, and comfortable ongoing interactions. Continuity of care increased many participants’ feelings of partnership with their doctor, with shared health goals, trust, and security. Participants without GP continuity were more resistant to engaging in GP care and expressed greater discomfort or dissatisfaction with the shared care model.

#### Health efficacy

Participants’ personal health efficacy facilitated their access to shared care, demonstrated by them taking an active role in their care. Capacity to self-advocate and seek access to better quality care following poor healthcare experiences, such as stigma or illness, were evidence of efficacy. Some attributed this to having high health literacy or feeling secure about their HIV status due to their length of time with the diagnosis or older age.

## Discussion

This study utilised a framework [21] to identify the determinants of access to shared care for PLWH in Northern NSW. The key factors affecting access were the perception of shared care providing additive value, the effect of past stigma on current healthcare engagement, and the compromise between quality care and logistical challenges. Overall, PLWH were satisfied with HIV specialist-led care but demonstrated mixed perspectives around increasing engagement with GPs.

Proactive access of care requires patients to perceive a benefit in accessing that service [37]. Overall, participants did not acknowledge or prioritise the potential positive health outcomes of shared care, and the role of GP care in this. Participants with long term specialist HIV care valued the social and interpersonal aspects of longer appointments and establishing trusting relationships with a specialist, which is also reflected in the literature [38]. GP consultations were generally viewed as lacking this valued interpersonal quality which led to shared care being perceived as lower value by some participants. This aligns with GPs’ perspectives that PLWH with prior long term specialist treatment appear to expect specialist-level care in GP environments [5], which is logistically unattainable in the GP setting. Participants with stable health and low comorbidity burden also perceived low need for shared care. While shared care is less indicated in this population, anticipating need may allow for the development of relationships prior to future health care crises and prevent the development of comorbid conditions.

Past experiences of stigma in healthcare have been found to cause subsequent healthcare avoidance or apprehension [2, 12, 16, 17, 19, 39, 40]. Participants perceived sexual health clinics as safe from stigma but GPs as relatively high risk. Trust and continuity of care were the most important facilitators of access in this context, particularly enabled by empathic and personalised consultation styles as well as flexible consultation lengths. In future shared care implementation, maximising continuity of care may be important in addressing this well established and widespread issue. Managing strict time constraints may be a future focus, having also been found to affect GP access for PLWH in other studies [18, 41]. In this study, participants felt less heard, less cared for and less likely to continue care when rushed in consultations. GPs have identified that longer consultation times facilitate healthcare in patients with anticipated stigma but pose a major challenge for financial feasibility [19]. Unlike the literature, fear of confidentiality breaches in GPs was not a primary barrier in this study as it has been identified elsewhere [5, 11, 12, 18]. This may be due to longer length of time living with HIV being related to reduced concerns regarding confidentiality [18]. Confidentiality may still be a prominent concern for PLWH outside of this sample.

Compromises between logistical considerations and quality GP care influenced access to GP care in this cohort. PLWH in rural NSW are more likely to have financial stressors [2] and travel greater distances for care [14]. Factors such as appointment availability, affordability and transport were important considerations for participants and eventuated in sacrificing care outright or being forced into lower quality care with poorer technical competency or interpersonal skills. Due to the importance placed on continuity of care in those with past stigma, lack of availability of a trusted GP was a deterrent for engaging in shared care and may lead to more anticipated or actualised stigma. In addition to compromised rapport, engaging in multiple GPs reduces effectiveness of shared care by complicating interprofessional communication pathways and coordination [5]*.* Low appointment availability, bulk billed and otherwise, is exacerbated by workforce limitations in rural NSW. Many participants financially required bulk billing practices; however, these were perceived as being more rushed, having less concern for holistic care and less continuity of care which was particularly disengaging in this patient group. Due to the low density of bulk billing practices and low availabilities, participants were travelling large distances and paying to access quality GP care.

As both technical and interpersonal competency were valued by participants, both must be addressed in increasing PLWH’s perceived need for shared care. Resistance to shared care has been shown to persist despite its advantages being identified [40], therefore patient education on the technical skills of GPs in primary care is insufficient in addressing this issue. The most significant factors to address in effective implementation of shared care are establishing trust and continuity of care for PLWH in general practice. Institutionalised stigma must be challenged in the training of current GPs and future medical practitioners to destigmatise care. A formal process to advertise HIV-friendly practices as an indicator of safe care for PLWH is recommended by this study and in accordance with Lazarus et al*.* [9]. To utilise the trusted healthcare relationship between PLWH and their HIV specialist, transition to shared care could be more actively managed by the specialist, including referral to reputably stigma-free GPs. This may also strengthen interdisciplinary relationships as smaller interprofessional communication networks have been shown to facilitate stronger coordination of shared care, particularly in low HIV caseload areas [5]. Furthermore, as availability of bulk billing is not ensured in rural Australia and PLWH are a vulnerable population, continuity of care could be assisted by government subsidies assisting non-bulk billing practices to provide GP care for PLWH. This could include financial incentives for extended consultation times which may benefit early rapport building and subsequent continuity. This population may also significantly benefit from the continuity of care outcomes predicted for voluntary patient registration in future GP reform [42].

Utilising the Levesque framework [21] was a strength of this study as it incorporated a holistic range of access determinants with clear definitions. Perspectives of people with long term HIV diagnoses gave insight into current impacts of access in the context of the evolution of HIV care. The generalisability of the data was affected by limited diversity of age and gender as studies have found that older people and men who have sex with men are more likely to have a regular GP than younger people and heterosexual people respectively [12], potentially overestimating GP engagement. This study was also limited by sampling as participants were primarily recruited from sexual health clinics and therefore did not represent some PLWH who solely receive GP care. Future research could focus on the implementation of targeted interventions addressing the challenges identified in this study, with a larger and more diverse sample of rural PLWH, including those receiving care only from GPs.

## Conclusion

Access to shared care is likely to be facilitated when GP engagement is perceived as providing additive value, both through health outcomes and rapport. Navigating anticipated stigma was a significant barrier to access and can be addressed by maximising trust and continuity within the logistical limitations of rural healthcare. While some action can be taken on the service level to reduce these barriers, strategies for system-wide changes could allow shared care to be utilised more effectively.

## Data Availability

The complete datasets supporting the conclusions of this article are not available openly due to the sensitive nature of the data and the consent provided for participation in the specific study. De-identified data can be requested from the corresponding author Dr Natalie Edmiston, noting that only fully de-identified and therefore limited data is available.

## Abbreviations

HIV: Human Immunodeficiency Virus
PLWH: People living with Human Immunodeficiency Virus
GP: General Practitioner
NSW: New South Wales
